# Who is lonely in lockdown? Cross-cohort analyses of predictors of loneliness before and during the COVID-19 pandemic

**DOI:** 10.1016/j.puhe.2020.06.036

**Published:** 2020-09

**Authors:** F. Bu, A. Steptoe, D. Fancourt

**Affiliations:** Department of Behavioural Science and Health, University College London, 1-19 Torrington Place, London, WC1E 7HB, UK

**Keywords:** Loneliness, mental health, Pandemic, COVID-19, Social isolation

## Abstract

**Background:**

There are concerns internationally that lockdown measures taken during the coronavirus disease 2019 (COVID-19) pandemic could lead to a rise in loneliness. As loneliness is recognised as a major public health concern, it is therefore vital that research considers the impact of the current COVID-19 pandemic on loneliness to provide necessary support. But it remains unclear, who is lonely in lockdown?

**Methods:**

This study compared sociodemographic predictors of loneliness before and during the COVID-19 pandemic using cross-cohort analyses of data from UK adults captured before the pandemic (UK Household Longitudinal Study, n = 31,064) and during the pandemic (UCL (University College London) COVID-19 Social Study, n = 60,341).

**Results:**

Risk factors for loneliness were near identical before and during the pandemic. Young adults, women, people with lower education or income, the economically inactive, people living alone and urban residents had a higher risk of being lonely. Some people who were already at risk of being lonely (e.g. young adults aged 18–30 years, people with low household income and adults living alone) experienced a heightened risk during the COVID-19 pandemic compared with people living before COVID-19 emerged. Furthermore, being a student emerged as a higher risk factor during lockdown than usual.

**Conclusions:**

Findings suggest that interventions to reduce or prevent loneliness during COVID-19 should be targeted at those sociodemographic groups already identified as high risk in previous research. These groups are likely not just to experience loneliness during the pandemic but potentially to have an even higher risk than normal of experiencing loneliness relative to low-risk groups.

## Highlights

• We compared data from 31,000 UK adults during 2017-2019 with 60,000 UK adults during the COVID-19 pandemic.
• Some risk factors for loneliness were the same as in ordinary circumstances (e.g. women and people living alone).
• Other groups experienced even greater risk of loneliness than usual (e.g. younger people and people of low income).
• Some groups were at risk of loneliness who are not usually considered high risk (e.g. students).

## Introduction

Loneliness has been recognised as a major public health concern associated with heightened risk of mental and physical illness, cognitive decline, suicidal behaviour and all-cause mortality.^1^, ^2^, ^3^ Loneliness itself has been referred to as an epidemic, and there have been heightened concerns about its effects during the global pandemic of coronavirus disease 2019 (COVID-19). Lockdowns and ‘stay-at-home’ orders announced internationally have led to physical and social distancing and reports of many individuals experiencing social isolation. Whilst social isolation (the absence of social interactions, contacts and relationships with others) is conceptually distinguished from loneliness (the feeling that one's social needs are not being met by the quantity or quality of one's social relationships), the two are known to be interrelated, with isolation often being a risk factor for becoming lonely.^4^ As a result, there have been calls to ascertain how the pandemic has affected loneliness to ensure that individuals at risk receive necessary support.^5^^,^^6^

In particular a key question is who is lonely in lockdown? On the one hand, individuals who already experience loneliness may be feeling even more isolated as a result of social distancing measures. Previous research has highlighted that particular groups at risk of loneliness include women, being either younger (e.g. aged younger than 25 years) or older (e.g. aged older than 65 years), living alone and having low socio-economic status, as well as poor mental and physical health.^7^^,^^8^ Preliminary research within Europe has suggested that these groups may indeed be at risk during lockdown and heightened loneliness is also affecting distress levels.^9^ However, it is also possible that enforced lockdowns are actually meaning that new groups are now at risk of loneliness.^10^ The pandemic has forced millions globally to curtail face-to-face contact and social activities, cut jobs and employment opportunities, restrict travelling and limit outdoor activity. For many individuals, this will be a radical departure from their patterns of usual daily life, and they may find habitual coping mechanisms (such as meeting with others) disrupted, leading to a heightened risk of feeling that the emotional and social support available to them is insufficient to meet their needs. It is important to understand predictors of loneliness during the pandemic even as first lockdowns ease because countries are likely to move in and out of further lockdowns over the coming months. Moreover, for some individuals at heightened risk of illness (‘shielding’), staying at home may be required until a vaccine is produced. Therefore, this study compared sociodemographic predictors of loneliness before and during the COVID-19 pandemic using cross-cohort analyses of data captured before and during the pandemic.

## Methods

### Participants

Data were drawn from two sources. For data collected before the pandemic, we used Understanding Society: the UK Household Longitudinal Study (UKHLS); a nationally representative household panel study of the UK population (2009–2019). Our analyses used the most recent wave of UKHLS (wave 9), where the loneliness measures were introduced. The wave nine data were collected between January 2017 and June 2019. To be consistent with the UCL COVID-19 Social Study, we restricted participants to those aged 18+, leaving us a total sample size of 34,976 participants. Furthermore, we excluded those who had missing value in loneliness or any of the covariates (11%). This provided a final sample size of 31,064.

For data during the COVID-19 pandemic, we used data from the UCL COVID-19 Social Study; a large panel study of the psychological and social experiences of more than 50,000 adults (aged 18+) in the UK. The study commenced on 21st March 2020 involving online weekly data collection from participants for the duration of the COVID-19 pandemic in the UK. Whilst not random, the study has a well-stratified sample that was recruited using three primary approaches. First, snowballing was used, including promoting the study through existing networks and mailing lists (including large databases of adults who had previously consented to be involved in health research across the UK), print and digital media coverage and social media. Second, more targeted recruitment was undertaken focusing on (i) individuals from a low-income background, (ii) individuals with no or few educational qualifications, and (iii) individuals who were unemployed. Third, the study was promoted via partnerships with third sector organisations to vulnerable groups, including adults with pre-existing mental illness, older adults and carers. The study was approved by the UCL Research Ethics Committee (12467/005), and all participants gave informed consent. In this study, we focused on participants who had a baseline response between 21st March and 10th May 2020. This provided us with data from 67,142 participants. Of these, 10% of participants withheld data on sociodemographic factors including gender and income and therefore were excluded, providing a final analytic sample size of 60,341.

### Measures

In both data sets, loneliness was measured using the three-item UCLA (University of California, Los Angeles) loneliness scale (UCLA-3). The questions are as follows: (1) how often do you feel a lack of companionship? (2) how often do you feel isolated from others? (3) how often do you feel left out? Responses to each question were scored on a three-point Likert scale ranging from hardly ever/never, to some of the time and to often. Using the sum score provided a loneliness scale ranging from 3 to 9, with a higher score indicating higher levels of loneliness. In addition, we also examined the single-item direct measure of loneliness asking how often the respondent felt lonely, which was coded on the same scale as the UCLA-3 items.

Covariates included age groups (18–29, 30–45, 46–59 and 60+), gender (woman vs. man), ethnicity (non-white vs. white), education (low: GCSE or below, medium: A-levels or equivalent, high: degree or above), low income (household annual income <£30,000 vs higher household annual income), employment status (employed, unemployed, student and inactive other), living status (alone, with others but no children, with others including children) and area of living (rural vs. urban). All variables aforementioned were harmonised between the two data sets.

### Analysis

To compare risk factors for loneliness, we used Ordinary Least Square regression models fitted separately in the two data sets. Survey weights were applied to both samples throughout the analyses to yield national representative samples of UK adults. The analyses of UKHLS used cross-sectional adult self-completion interview weights, whereas analyses of the UCL COVID-19 Social study were weighted to the proportions of gender, age, ethnicity, education and country of living obtained from the Office for National Statistics.^11^ The descriptive and regression analyses were implemented in Stata v15 (StataCorps, Texas).

## Results

Descriptive statistics for the two samples are shown in Table 1. Loneliness levels were higher in the UCL COVID-19 Social Study than in UKHLS, with 32.5% of people feeling lonely sometimes (28.6% in UKHLS) and 18.3% often (8.5% in UKHLS).

**Table 1:** Descriptive statistic of the explanatory variables (weighted).

Variables	Categories	UKHLS (N = 31,064)	Covid-19 Social Study (N = 60,341)
Age	18–29	16.8%	18.5%
	30–45	23.1%	27.2%
	46–59	25.5%	24.4%
	60+	34.7%	29.9%
Gender	Women (vs. men)	51.8%	49.8%
Ethnicity	Non-white (vs. white)	7.2%	12.5%
Education	GCSE or below	50.6%	32.0%
	A-levels or equivalent	23.2%	33.5%
	Degree or above	26.2%	34.5%
Household income	Low (<30 k) (vs. high)	40.1%	48.6%
Employment status	Employed	56.6%	59.6%
	Unemployed	3.8%	3.3%
	Student	3.6%	6.5%
	Inactive other	36.0%	30.6%
Living status	Alone	18.9%	18.3%
	With others (not children)	49.9%	53.9%
	With others (including children)	31.2%	27.8%
Area of living	Rural (vs. urban)	24.5%	20.4%
UCLA loneliness scores	UCLA-3: score 3	48.4%	34.0%
	UCLA-3: score 4	13.9%	13.8%
	UCLA-3: score 5	11.8%	12.9%
	UCLA-3: score 6	15.7%	17.0%
	UCLA-3: score 7	4.0%	7.6%
	UCLA-3: score 8	2.6%	5.9%
	UCLA-3: score 9	3.5%	8.8%
How often do you feel lonely	Hardly ever/never	62.9%	49.2%
	Sometimes	28.6%	32.5%
	Often	8.5%	18.3%

UCLA, University of California, Los Angeles; UKHLS, UK Household Longitudinal Study.

**Fig. 1:**
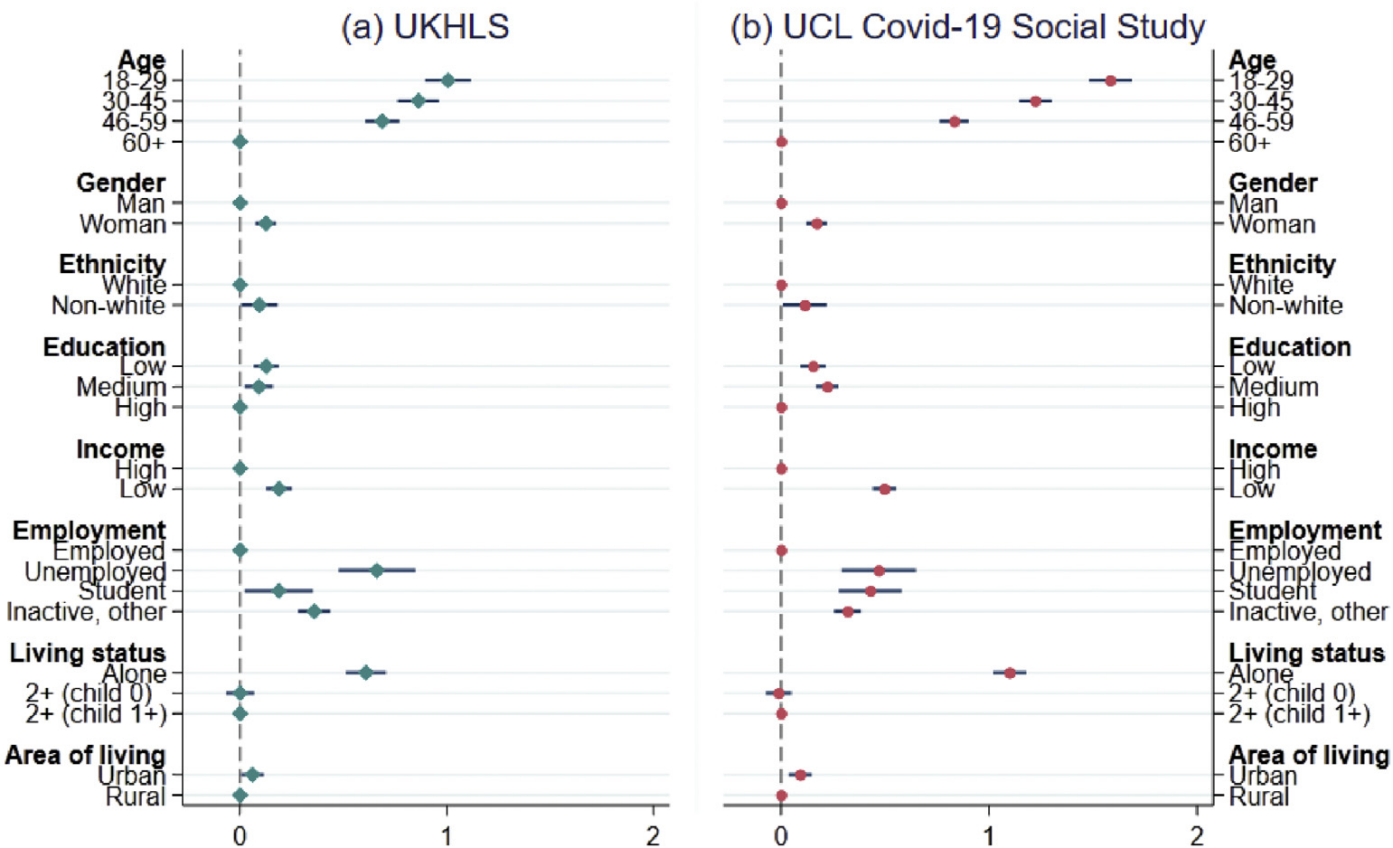
Coefficients and 95% confidence intervals from the regression model on loneliness.

Risk factors for loneliness were near identical before and during the pandemic. Fig. 1 presents the estimated coefficients (coef) and 95% confidence intervals (CI) from the regression models. Adults aged 18–30 years were more likely to be lonely compared with adults aged 60+ before the pandemic (coef = 1.01, 95% CI: 0.89–1.12), and during the pandemic (coef = 1.58, 95% CI: 1.48–1.68). People living alone, similarly, were more at risk before and during the pandemic (coef = 0.61, 95% CI: 0.51–0.71 vs coef = 1.10, 95% CI: 1.02–1.18). Having a low household income and being unemployed were also persistent risk factors. Being a student was only a moderate risk factor before the pandemic (coef = 0.19, 95% CI: 0.02–0.35) but was a greater risk factor during the pandemic (coef = 0.43, 95% CI: 0.28–0.58). Other risk factors including non-white ethnicity, being a woman, having low educational attainment and living in urban areas were only small risk factors but were maintained before and during the pandemic.

## Discussion

This study explored who was most at risk of loneliness during the UK lockdown due to the COVID-19 pandemic and compared whether risk factors were similar to risk factors for loneliness before the pandemic. Young adults, people living alone, people with lower education or income, the economically inactive, women, ethnic minority groups and urban residents had a higher risk of being lonely both before and during the pandemic. These results echo previous studies on risk factors for loneliness.^7^^,^^8^ These findings in the UK are also echoed by some recent data from Spain during their lockdown, which highlighted similar risk factors.^9^ However, these data show that some people who were already at risk for being lonely (e.g. young adults aged 18–30 years, people with low household income and adults living alone) experienced an even greater risk during the COVID-19 pandemic compared with usual (indicated by higher coefficients). Furthermore, being a student emerged as a higher risk factor during lockdown than usual, although this builds on wider research suggesting that loneliness can be a problem for students and has been rising over the past six years.^12^

This study has a number of strengths including its cross-cohort comparisons of two large samples with harmonised measures before and during the pandemic, as well as its consideration of a broad range of sociodemographic characteristics. However, the data compared are from different participants, hence it is not clear whether those individuals experiencing loneliness during lockdown had previous experience of loneliness. Furthermore, the COVID-19 Social Study is a nonrandom (albeit large, heterogeneous, well-stratified and weighted) sample. Hence the results presented here are not presented as accurate prevalence figures for loneliness during the pandemic. It is possible that the study inadvertently attracted individuals who were feeling more lonely to participate. Finally, the study looked at broad risk categories. Future studies are encouraged to (i) consider whether the interaction between different risk categories (e.g. unemployed adults living alone) or accumulation of multiple risk factors affected loneliness levels during the pandemic, (ii) track the trajectories of loneliness across lockdown and (iii) explore the potential buffering role of protective social or behavioural factors.

Overall, these findings suggest that interventions to reduce or prevent loneliness during COVID-19 should be targeted at those sociodemographic groups already identified as high risk in previous research. These groups are likely not just to experience loneliness during the pandemic but to have an even higher risk than normal of experiencing loneliness relative to low-risk groups. Such efforts are particularly important, given rising concerns that loneliness could exacerbate mental illness and lead to non-adherence to government regulations.^13^^,^^14^ As such, supporting individuals experiencing loneliness during and in the aftermath of the pandemic should be a public health priority.

## Author statements

### Ethics approval

Ethical approval for the COVID-19 Social Study was granted by the UCL Ethics Committee. All participants provided fully informed consent. The study is GDPR compliant.

### Competing interests

All authors declare no conflicts of interest.

### Funding

This Covid-19 Social Study was funded by the 10.13039/501100000279Nuffield Foundation [WEL/FR-000022583], but the views expressed are those of the authors and not necessarily the Foundation. The study was also supported by the MARCH Mental Health Network funded by the Cross-Disciplinary Mental Health Network Plus initiative supported by 10.13039/100014013UK Research and Innovation [ES/S002588/1], and by the 10.13039/100010269Wellcome Trust [221400/Z/20/Z]. D.F. was funded by the 10.13039/100010269Wellcome Trust [205407/Z/16/Z]. The researchers are grateful for the support of a number of organisations with their recruitment efforts including: the UKRI Mental Health Networks, Find Out Now, UCL BioResource, HealthWise Wales, SEO Works, FieldworkHub, and Optimal Workshop. The funders had no final role in the study design; in the collection, analysis and interpretation of data; in the writing of the report; or in the decision to submit the article for publication. All researchers listed as authors are independent from the funders, and all final decisions about the research were taken by the investigators and were unrestricted. All authors had full access to all of the data (including statistical reports and tables) in the study and can take responsibility for the integrity of the data and the accuracy of the data analysis.

### Availability of data and materials

Anonymous data will be made available after the end of the pandemic. Full details of the COVID-19 Social Study including a the study protocol and user guide are provided at www.COVIDSocialStudy.org.

### Authors’ contributions

F.B., A.S. and D.F. conceived and designed the study. F.B. analysed the data, and F.B. and D.F. wrote the first draft. All authors provided critical revisions. All authors read and approved the submitted manuscript.
